# On the Pathology of Spontaneous Hæmorrhage

**Published:** 1852-07

**Authors:** A. B. Palmer


THE NORTH-WESTERN

MEDICAL AND SURGICAL JOURNAL.

NEW SERIES.

VOL. I. JULY, 1852. No. 3.

### ORIGINAL COMMUNICATIONS.

ARTICLE I.—*On the Pathology of Spontaneous Hæmorrhage.* An Essay read before the Chicago Medical Society, February, 1852, by A. B. PALMER, M.D.

[CONCLUDED.]

III. IN the 3d place we are to notice the deviation from the natural standard of the *forces with which the blood is impelled* as a cause of hæmorrhage.

This deviation of the impulsive force may exist in the heart and the general circulation,—or it may be limited to the localities from which the hæmorrhage occurs.

Among the conditions of the general circulation predisposing to hæmorrhage, are hypertrophy of the heart—particularly of the left side,—disease of the valves, or any cause which shall obstruct the blood through the right side of the organ,—increased action of the heart from the too stimulating qualities of the blood, as is the case in a superabundance of the red globules,—or increased action of the general circulatory system from stimulating drinks—from abundance of stimulating food—from active exercise—febrile excitement—mental emotions—nervous irritability, or whatever other cause which produces that effect.

It is particularly worthy of remark that in many cases of hæmorrhage, or where hæmorrhage is threatened, there is a peculiar irritability of the whole circulatory system, giving a peculiar jerking thrill to the pulse. This condition seems to consist in an unusual abruptness of the heart's contractions, combined with irregularities in the tonicity of the arteries in different parts, which cause them to react in successive jerks at each pulsation, instead of simultaneously and uniformly.

This condition of things may be considered as a dividing link between the general disturbances of the circulating force and those deviations of a local character.

These local deviations of the circulating force are produced by a variety of causes, among which are *mechanical means*—as, for instance, position; the law of gravitation modifying the ordinary circulating force. Examples of this are found in epistaxis and apoplexy from stooping, uterine hæmorrhages from rising, &c.

Lifting and straining affect the circulatory force in particular localities, partly by increasing the general action of the heart and arteries, and partly by retarding by muscular pressure the return of blood from those localities through the veins.

*Variations of temperature* vary the circulation; as cold applied to some parts while others are at a higher temperature.

Local irritants vary the circulation in the parts to which they are applied, disposing to hæmorrhage.

But a more frequent cause of hæmorrhage arising from deviations in the circulating force of a local character, exists in what is called "determinations of blood," or *local hyperæmia.*

Increased quantity of blood in a part is produced by the causes just enumerated; but this we are now considering exists independently of any of these more obvious causes.

This condition essentially consists in a spontaneous enlargement of the arteries of a part, produced by some vital alteration of their tonicity, the blood being propelled into them by the *vis a tergo.* At least this is the explanation confidently set forth by some of our most philosophical writers. But the inquiry may with propriety be made, whether in these cases there is not a decidedly increased active expansive and contractile force in the vessels of the part, or some change in the vital affinities of the blood, the vessels, the nerves and other tissues, of a more positive character, and not to be accounted for upon the simple notion of passive dilatation of the smaller vessels. Although these points are all involved in obscurity, many of the remote excitants of these "determinations"—though we shall not now refer to them—are better understood.

Hæmorrhages from this cause are usually preceded by increased heat, redness and throbbing in the part—indeed, by many of the phenomena which precede an attack of inflammation.

The manner in which increased circulatory force, whether general or local, operates in inducing hæmorrhage, is too obvious to require illustration or remark.

We have now considered as fully as present time will justify, the different pathological elements which dispose to, or produce, spontaneous hæmorrhage. As already intimated, in most of the cases of actual hæmorrhage which occur, several of the elements we have enumerated combine to produce the result, and to some of these combinations, as they exist in particular forms of the disease, we will briefly allude.

In ordinary cases of true hæmorrhagic apoplexy in sanguineous persons, the disease is produced by the combined influence of an abnormal condition of the blood and of the propelling force. The blood is too great in quantity and has more red corpuscles and less fibrin than in health, and the manner in which these conditions tend to produce hæmorrhage has already been explained. But perhaps the disease depends still more upon a morbid condition of the propelling forces. There is a general increase of the circulatory powers produced by the stimulating qualities of the blood exciting the heart and arteries; this effect, increased, of course, by any transient cause of excitement of the general system—and the results of these causes are still further increased by the determination of blood to the part,—from the various causes acting on the nervous system, or on mechanical principles.

In the cases here referred to, of course rupture of the vessels occurs as the immediate cause of the extravasation of blood. It will be observed in these, as in many other cases, there is a chain—a succession of causes and consequences extending from the remotest cause to the final result.

In scurvy, the condition of the blood has probably less to do in producing hæmorrhage, where that occurs, than is generally supposed. Having referred already to this disease in illustrating another point, we may here simply remark that fibrin is abundant and the blood coagulates readily, and the red corpuscles are slightly deficient; but, as before stated, there may be some abnormal condition of the blood—a change in the *qualities* of some of its constituents, though not in their proportions, favoring hæmorrhage, which is not, in the present state of our knowledge, quite appreciable. However, there can be little doubt that the chief difficulty in this disease is in the solids—in the tissues of the blood vessels, which are less firm and consistent. By a loss of tonicity the capillaries are distended, and the ordinary force of the circulation presses the blood through, rupturing the brittle vessels even where no ulcerative process exists.

In ordinary cases of acute purpura hæmorrhagica, to which reference has also been made, the proportion of fibrin and corpuscles to each other and to the rest of the blood is nearly natural; and the coagulability of the fibrin is not materially impaired. But here it is probable that changes occur in the quality of these ingredients, the precise character of which is not ascertained. It is supposed by some, and not without much plausibility, that in the case of purpura, and in some other instances of hæmorrhagic diseases, morbid and foreign materials exist in the blood, modifying the condition of its natural elements; and this view in regard to purpura hæmorrhagica—bile being regarded as that element—is strengthened by facts which have long been observed, and are clearly ascertained. It is known as the result of direct experiment out of the body, that bile exerts a solvent influence upon blood-corpuscles. That it produces a similar effect within the body is inferred from the fact that in fatal cases of jaundice connected with structural disease of the liver, large spots of ecchymosis have frequently been observed upon the surface, apparently the result of the effusion into the tissue of dissolved hæmatine. It is further known that in purpura the action of the liver is generally decidedly diminished, and the disease is most speedily relieved by addressing remedies to that condition of the organ. This train of facts seems to indicate that in purpura, although the elements of the blood are in their normal proportions, one of them—the blood corpuscles—is changed, more or less of it dissolved by the bile—facilitating its passage out of the vessels and its effusion into the tissues; but still, here, as in the case of scurvy, in the absence of positive knowledge we must attribute the fault, in a very large degree at least, to the minute blood vessels which, either from structural change, become less tenacious, or from a deficiency of vital tonicity, yield to the force with which the blood is propelled and discharge their contents from a solution of their continuity.

In petechial fevers, hæmorrhagic small pox, and other hæmorrhagic exanthemata, the blood, though abounding in red particles (somewhat changed, however), is deficient in fibrin; and this condition of the fluid combined with a similar defect in the texture of the vessels as in the cases before mentioned, and these conditions still further combined with a disturbed state of the general and local circulation, produce the symptom.

In hæmorrhages occurring in cases of general mal-nutrition, cachectic and depraved conditions of the system, the blood, the tissues and the circulating forces, all are more or less in fault,—varying in their proportions in each particular case.

In pathology, as in many other things, "extremes often meet," i. e., opposite conditions produce similar effects, and this principle is illustrated in the fact that an extreme impoverishment and deficiency of the blood produces an analogous effect—excites the heart and arteries in a similar manner as an over-richness and superabundance of that fluid does. I do not intend to say that the effects of anæmia and spanæmia upon the circulating forces are identical with those of hyperæmia; but in both instances an increased action is likely to be produced, tending to hæmorrhage. In a given action and power of the circulating force, with a given bulk of blood in the system, a spanæmic, or thin and impoverished state of the fluid, must be much more favorable to the production of hæmorrhage, than the opposite condition, and we find that some of the most profuse and alarming hæmorrhages occur under these circumstances.

In the spontaneous hæmorrhages which occur in persons suffering from the poison of the rattle-snake, it is highly probable that a changed condition of the blood is a principal cause. Observations have shown that many of the blood disks are broken down and the hæmatin is held in solution; but still the tissue of the vessels is doubtless also impaired.

Upon the subject of the essential pathology of what is called

"the hæmorrhagic diathesis," or that constitutional peculiarity of some persons to bleed profusely from the slightest wound, though in other respects in health, I have sought in vain for light. I have found no record of any microscopical or chemical examination of the blood in these subjects, or of any minute examination of the condition of the vessels. If such investigations have been made and recorded they have not fallen under my observation, and I have taken some pains to search.

We do not learn from the histories of these cases, as contained in the books and scattered through numerous journals, that hæmorrhage is particularly likely to occur of a strictly spontaneous character, or unless a wound is made. From this circumstance, and the otherwise good health of those persons, we would rather be led to the conclusion that the blood could not be seriously in fault, and that the *texture* of the vessels could not be specially defective, but rather that they were abnormal in their *action*—not possessed of that usual contractility by which quality nature principally operates in arresting the flow of blood from divided vessels. These curious cases possess no small amount of interest, and will doubtless ere long be submitted to that careful and rigid scrutiny which every subject connected with our profession is, with the advancement of science, destined to undergo.

In the different varieties of uterine hæmorrhage, including menstruation, the cause exists rather in the circulating forces and in the physical condition of the vessels than in the blood. Any existing abnormal condition of the blood may of course contribute to the result; but in a large majority of cases no such condition exists. There is usually either a lesion of the vessels as a primary condition, or such a state of the local circulating forces as to produce such lesion as a secondary consequence, which in turn becomes the immediate *cause* of the flow.

Other particular forms of hæmorrhage might be referred to, and the special pathology of each more elaborately considered; but observations respecting these varieties have been sufficiently extended to illustrate the views taken; and the applicability of our system to all cases will readily be seen.

In looking over the list of particular forms of hæmorrhage it will be found that those from the mucous membrane are by far the most numerous, and most frequently occurring. The greater susceptibility of this membrane to pour out blood, over the other surfaces of the body, arises from its peculiar structure, viz.: its greater vascularity, the delicacy of its epithelial covering, the loose manner in which it is connected with the parts underneath it, and the pores which permit the passage of mucus are doubtless larger than those which permits the passage of the thinner fluids, as serum and perspirable matters.

A full discussion of the characters of hæmorrhage would lead to a consideration of its classifications into active and passive, critical, periodical, vicarious, &c., but this paper does not claim to be a complete treatise; and besides the essential pathology of all these varieties is, in a general way, embraced under the different heads into which we have already divided the subject. The books treating upon the pathology of hæmorrhage, to which I have had access, are confused and vague, and, at least to my mind, quite unsatisfactory; and if the foregoing views shall serve as hints to more precise, definite and rational notions; or lead to a more philosophical mode of conducting investigations upon the subject, a far greater object than was anticipated when the task was undertaken, will have been accomplished.

